# Phenotypic and transcriptional response of *Daphnia pulicaria* to the combined effects of temperature and predation

**DOI:** 10.1371/journal.pone.0265103

**Published:** 2022-07-14

**Authors:** Aaron Oliver, Hamanda B. Cavalheri, Thiago G. Lima, Natalie T. Jones, Sheila Podell, Daniela Zarate, Eric Allen, Ronald S. Burton, Jonathan B. Shurin

**Affiliations:** 1 Center for Marine Biotechnology and Biomedicine, Scripps Institution of Oceanography, University of California, San Diego, La Jolla, California, United States of America; 2 Department of Biological Sciences, Ecology Behavior and Evolution Section, University of California, San Diego, La Jolla, California, United States of America; 3 Marine Biology Research Division, Scripps Institution of Oceanography, University of California, San Diego, La Jolla, California, United States of America; University of Shiga Prefecture, JAPAN

## Abstract

*Daphnia*, an ecologically important zooplankton species in lakes, shows both genetic adaptation and phenotypic plasticity in response to temperature and fish predation, but little is known about the molecular basis of these responses and their potential interactions. We performed a factorial experiment exposing laboratory-propagated *Daphnia pulicaria* clones from two lakes in the Sierra Nevada mountains of California to normal or high temperature (15°C or 25°C) in the presence or absence of fish kairomones, then measured changes in life history and gene expression. Exposure to kairomones increased upper thermal tolerance limits for physiological activity in both clones. Cloned individuals matured at a younger age in response to higher temperature and kairomones, while size at maturity, fecundity and population intrinsic growth were only affected by temperature. At the molecular level, both clones expressed more genes differently in response to temperature than predation, but specific genes involved in metabolic, cellular, and genetic processes responded differently between the two clones. Although gene expression differed more between clones from different lakes than experimental treatments, similar phenotypic responses to predation risk and warming arose from these clone-specific patterns. Our results suggest that phenotypic plasticity responses to temperature and kairomones interact synergistically, with exposure to fish predators increasing the tolerance of *Daphnia pulicaria* to stressful temperatures, and that similar phenotypic responses to temperature and predator cues can be produced by divergent patterns of gene regulation.

## Introduction

Many species are at risk of extinction as the environment changes at an unprecedented pace. Ecological and evolutionary research aims to predict species’ responses to anthropogenic environmental change, such as global warming and introduction of predators [1, 2]. To cope with such stressors, species can either migrate to more suitable habitats or adapt to new conditions. Understanding the potential and limits of genetic adaptation and phenotypic plasticity to maintain fitness is critical in order to predict persistence of populations and changes in biodiversity under environmental stresses [3]. The mechanisms by which organisms are adapted to present day conditions throughout their ranges therefore provide a basis for predicting extinction, persistence, and changes in biodiversity in a rapidly changing environment [4, 5].

Plasticity and genetic adaptation impact organismal fitness in response to environmental change. Plasticity allows a genotype to express multiple phenotypes in different environments whereas genetic adaptation arises from environment-dependent variation in fitness of different genotypes [6]. Evidence for rapid evolution in natural systems, particularly in response to human-induced environmental change, indicates that most of the observed changes are not genetically based, but rather a consequence of plasticity [7]. Plasticity at level of gene expression is one of the most important mechanisms for coping with stress [8, 9], yet the magnitude and genetic basis of such plasticity remains largely unknown.

Plasticity arises from differential gene expression patterns in response to environmental cues [10]. Variable levels of plasticity may evolve in a population if reaction norms differ across genotypes and the slope of the reaction norm is correlated with fitness [11]. Either decreases or increases in phenotypic plasticity could contribute to adaptation to variable environments depending on the rate and predictability of environmental change [6, 12].

Several factors could constrain the evolution of plasticity at the transcriptome level. First, genetic variation potential for plasticity could be limited or absent. Alternatively, even when genetic variation is present, its evolution could be constrained by costs of the mechanisms underlying plastic responses [13]. A trade-off is expected where enhanced plasticity would be beneficial in more spatially or temporally variable environments, but detrimental in a stable environment [14, 15]. In addition, if environmental variation is unpredictable, then plasticity may fail to match phenotypes to the environment to produce higher fitness.

*Daphnia* is an ecologically important genus of crustacean zooplankton in lakes that transfers energy from phytoplankton to fish and invertebrate predators and exerts top-down grazing control on primary production [16]. *Daphnia* shows both genetic adaptation and plasticity at a phenotypic level in tolerance to two important determinants of fitness: temperature and predation. Previous studies have documented that the response of *Daphnia* populations to thermal stress is strongly correlated with environmental conditions and can be either genetic, plastic or both [17–19]. Although these studies show plastic and evolutionary changes, the co-occurrence of other environmental stressors, such as predation, with temperature might interact in natural populations. Fish predation and temperature impose selection on many of the same traits, and in the same direction [18, 20]. For instance, *Daphnia* often mature at smaller sizes and younger ages in warmer water and when fish are present, yet little is known about the molecular basis of this response among populations or their potential interactive effects.

We collected *Daphnia pulicaria* from two lakes in the Sierra Nevada Mountains, California, USA with different thermal conditions to conduct an experiment to evaluate the degree to which life history, thermal tolerance and gene expression are influenced by temperature and predation. We predicted that warm temperatures and exposure to predators should produce similar responses in life history traits, then used transcriptomics to ask whether the same genes underlie the plastic response to fish kairomones and warming, or if the similar phenotypic effects arise from pleiotropic effects where altered gene regulation affects multiple phenotypic traits. Our goal was to ask whether *D*. *pulicaria’s* plastic response to one selective agent (fish or warming) magnified or dampened the response to the other in terms of both phenotype and gene expression.

## Materials and methods

### Lake sampling

Gardisky (37.955774, -119.251198) and Blue Lakes (38.051164, -119.270342), located in Inyo National Forest, California, USA, were sampled in late August and early September 2017, respectively. In both lakes we measured temperature throughout the water column at each meter using a field probe (YSI Incorporated, Yellow Springs, Ohio, USA). We also collected live zooplankton from the deepest point of the lake using a 30 cm diameter, 63 μm mesh conical net with a 1 m length through the water column, starting 1 m above the lake bottom. These samples were kept cold until returning to the laboratory, where we searched for *Daphnia pulicaria*. When present, 30 *D*. *pulicaria* females carrying eggs in the brood pouch were separated in 50 ml centrifuge tubes filled with COMBO medium [21]. Each *Daphnia* individual was considered a maternal line. Each maternal line was cultured for at least twelve generations in separate 50 mL tubes filled with COMBO medium under ambient lab conditions. All *D*. *pulicaria* were fed non-viable cells of the green alga *Nannochloropsis* sp. (Brine Shrimp Direct, Ogden, Utah, USA) at a constant high rate of 24 × 10^6^ cells per 50 mL tube every two days.

### Experimental design

We started by randomly picking one mature female from each clone. Upon the release of the second clutch, we isolated three neonates that were separated and moved to separate 100 mL containers containing the same media and algae. All individuals were transferred to fresh media and algae three times a week and reared at 15 ± 1°C and photoperiod 12:12 h light/dark. Propagation continued until 100 neonates were collected for each of the clonal lines, with all individuals used for further study born within a 12-hour period. All neonates were placed into 100 ml jars containing COMBO medium at a density of 3 *Daphnia*/jar [21]. Each jar was randomly allocated to one of four treatments: (1) optimum temperature (15°C) without kairomones (fish cues), (2) high temperature (25°C) without fish cues, (3) optimum temperature with fish cues, and (4) high temperature with fish cues. An optimum temperature near 15°C is consistent with previous reports of thermal preference in *Daphnia pulicaria* [22], although there is variation in tolerable temperatures between *D*. *pulicaria* genotypes [23]. All *Daphnia* were transferred to fresh medium, with algae (and kairomones in the fish cue treatment) daily. We monitored jars daily for maturation (i.e., release of first clutch into the brood chamber). Upon reaching maturity, individuals were either preserved in RNAlater (Qiagen, USA) and kept in -20°C for subsequent RNAseq analyses or assigned to phenotypic assay (see below). RNA was extracted from whole Daphnia individuals. No food was added during the last 12 hours before sampling to minimize algal RNA contamination, as most algae will be digested and degraded after 12 hours. The period without food was kept short to minimize starvation-dependent gene regulation.

### Kairomone collection

COMBO medium conditioned by the presence of planktivorous fish was collected daily from a tank containing 5 juvenile rainbow trout (*Oncorhynchus mykiss*; ~ 5 cm in tail-to-snout length per juvenile) in 72 L of COMBO. Each day, media containing fish chemical cues was filtered through 0.7 μm mesh membrane filters and added at a concentration of 0.007 fish/L to the predator treatments. This concentration of kairomone media is above the threshold used by other studies to elicit a phenotypic response, such as the concentration of 5 μl of kairomone media per 100 ml of solution [24].

*D*. *pulicaria* predation by *O*. *mykiss* in artificially stocked lakes is well established, specifically predation by juvenile rainbow trout [25, 26]. *O*. *mykiss* is one of the most abundant species for stocking previously fishless lakes in the Sierra Nevada mountains [29], and *O*. *mykiss* individuals were observed in both Blue and Gardisky Lake during sampling. *O*. *mykiss* were fed TetraMin Tropical Flakes (Tetra, Blacksburg, Virginia, USA) prior to kairomone collection, rather than juvenile conspecific daphniids as in some other studies [27, 28]. We selected this method to minimize the presence of conspecific alarm cues in the isolated kairomone media, so that we could measure the phenotypic and genetic responses to only predator-released, diet agnostic kairomone cues. Such research strategies are supported by Mitchell et al. [29], who call for further research to determine the relationship between predator odor and diet-based cues in aquatic systems. Following their nomenclature recommendations, we define the kairomones used in this study to be predator odor cues, as opposed to alarm cues released by injured conspecifics or diet cues released by predators after digesting conspecifics. All procedures involving animals were reviewed and approved by the Institutional Animal Care and Use Committee of the University of California San Diego (IACUC protocol number: S14140).

### Phenotype assays

Individuals from the experimental generation assigned to the phenotypic assay were scored for the following life-history variables: age and size at maturity, and maternal age at the release of each of the first three clutches and the number of offspring in these clutches. These data were used to calculate intrinsic population growth rate for each maternal line following the Lotka–Euler equation [30]. We also measured critical maximum temperature (CT_max_) using a heat ramping assay. After a 30-minute resting period at ambient temperature, *Daphnia* from the four treatments were transferred to 0.5ml Eppendorf tubes and placed in a thermal heater (4x6 thermal heater, Corning Digital Dry Bath Heater Dual Block). The water temperature increased 0.1°C every 20 seconds. We continuously monitored the state of individuals and recorded the temperature when each individual *Daphnia* lost swimming ability and sank to the bottom of the tube. The temperature when *Daphnia* became immobilized was used as a proxy for CT_max_ [17]. We measured life history traits of 11 to 13 individuals per treatment per clone and CT_max_ of 10 to 12 individuals per treatment per clone. In total, we scored phenotypes of 186 individuals.

### Statistical analysis for phenotype

We log-transformed all continuous variables, except intrinsic growth rate, after visually assessing the probability distribution that best fit the data. We analyzed the effects of the treatments using general linear mixed-effect models for each trait using the lmer() function of the R package lme4 v. 1.1.27.1 [31]. Temperature treatment, fish cue treatment, and the interactions of these variables were modeled as fixed effects for age and size at maturity, average number of offspring, intrinsic growth rate and CT_max_. Clone, or source lake, was considered a random effect. Since we expected that CT_max_ would be influenced by body size we included an additive effect of size.

Additionally, to test the effects of slopes of the reaction norms, we calculated the pairwise differences for each dependent variable between each treatment combination within a clone. We computed pairwise differences using the least square mean values based on the general linear models for each trait using *lsmeans* function in the *lmerTest* R package [32]. The pairwise difference test was corrected using Tukey’s adjustment for multiple comparisons [33].

### RNA isolation, library preparation and sequencing

We extracted RNA using the TRI Reagent Protocol (Sigma-Aldrich, USA) from samples preserved in RNAlater (Qiagen, USA). Any remaining genomic DNA was removed using the TURBO DNA-free Kit (Invitrogen, USA). Each treatment included five biological replicates, with each replicate comprised of 35 clonal *Daphnia* individuals. Extracted RNA was stored in -80°C until sequencing. Quality of the isolated RNA was assessed using RNA Nano 6000 Assay Kit of the Agilent Bioanalyzer 2100 system (Agilent Technologies, CA, USA) and mRNA-seq libraries were constructed using NEBNext UltraTM RNA Library Prep Kit for Illumina (NEB, USA) following manufacturer’s recommendations. A total amount of 1 μg RNA per replicate was used as input material for RNA-seq library preparations. Samples that did not reach 1 μg RNA were pooled with a biological replicate grown under the same conditions, a process described by Takele Assefa et al. [34], leaving 18 replicates from Blue Lake and 16 replicates from Gardisky Lake. Index codes were added to attribute sequences to each sample and all 34 libraries were clustered on a cBot System using the PE Cluster kit cBot-HS (Illumina). After generating the clusters, libraries were sequenced using the Illumina HiSeq 2000 platform and 150 bp paired-end reads were generated.

### Read cleaning and transcriptome comparison

The raw reads were preprocessed using fastp v. 0.23.1 [35] with the command line options “-x -f 18 -F 18”. These options enabled polyX tail trimming and removed 18 low-quality bases from the beginning of all forward and reverse reads, respectively. To check for contamination in our read set, we used Kraken2 v. 2.0.9 [36] with a taxonomy database constructed from the NCBI nr database [37] as of August 2021. A random subset of 1,000,000 read pairs was used for each Kraken2 run per sample. All downstream analyses were based on the remaining cleaned, validated data.

Separate *de novo* assemblies for were performed on the Blue and Gardisky samples using Trinity v. r20140413p1 [38] with parameter min_kmer_cov set to 2. Transcriptome completeness was assessed using BUSCO v. 5.2.2 [39] with the arthopoda_odb10 lineage dataset, and also by comparison to the following published, annotated genomes: *D*. *pulicaria* LK16 [40], *D*. *pulex* TCO [41], *D*. *galeata* M5 [42], *D*. *magna* SK [43] and *Eulimnadia texana* JT4(4)5-L [44].

### Differential expression analysis

Relative expression levels of annotated genes were estimated by independently mapping RNA-Seq reads from each clone back to the annotated *D*. *pulicaria* LK16 gene set using STAR v. 2.7.9a [45]. A read count matrix was generated from these alignments using featureCounts v. 2.0.1 [46]. Genes with read counts below 10 across all conditions were filtered from the remaining pipeline. The significance of difference in gene expression of each treatment for each genotype was determined with the R package DESeq2 v. 1.32.0 [47], using a fold change test based on negative binomial distribution. Statistical significance (p-value) was adjusted using the q-value obtained from the false discovery rate [48], with a q-value < 0.05 and |log2(foldchange)| > 1 set as the threshold for significant differential expression. Counts of differentially expressed genes across treatments were visualized using the R package VennDiagram v. 1.7.1 [49]. Read counts corrected through the variance stabilizing transformation [50] function of DESeq2 were visualized using a principle component analysis (PCA). The PCA was generated using corrected read counts from every gene, rather than the DESeq2 default of using only the 500 most variable genes. These corrected read counts were also used to perform a PERMANOVA test [51] through the R package vegan v. 2.5–7 [52] with 999 permutations. The groups tested with PERMANOVA were based on clone, temperature, fish cues, and their interactions terms.

Gene Ontology (GO) enrichment analyses were performed to determine whether significantly differentially expressed gene sets were enriched for any biologically relevant functional categories [53]. GO term enrichment lists were generated using GOseq v. 1.44 [54] for the following one-factor comparisons: Blue, temperature rise with kairomones; Blue, temperature rise without kairomones; Gardisky, temperature rise with kairomones; Gardisky, temperature rise without kairomones. Further control of the False Discovery Rate was performed using the Benjamini–Hochberg method [55], and the cutoff for enriched GO terms was an adjusted p-value less than 0.05.

### Orthology of differentially expressed genes

*Daphnia pulex* genes previously demonstrated to be differentially expressed in response to temperature and/or predator kairomones were accessed from the *Daphnia* Stressor Database [56] as of September 2021. Predicted protein sequences for these genes were downloaded from wFleaBase [57]. These genes were compared to the annotated *Daphnia pulicaria* LK16 genome using OrthoVenn2 [58] with an e-value of 1e-5. OrthoVenn2 results were visualized using the R package VennDiagram v. 1.7.1 [49].

## Results

### Phenotype

**Fig 1:**
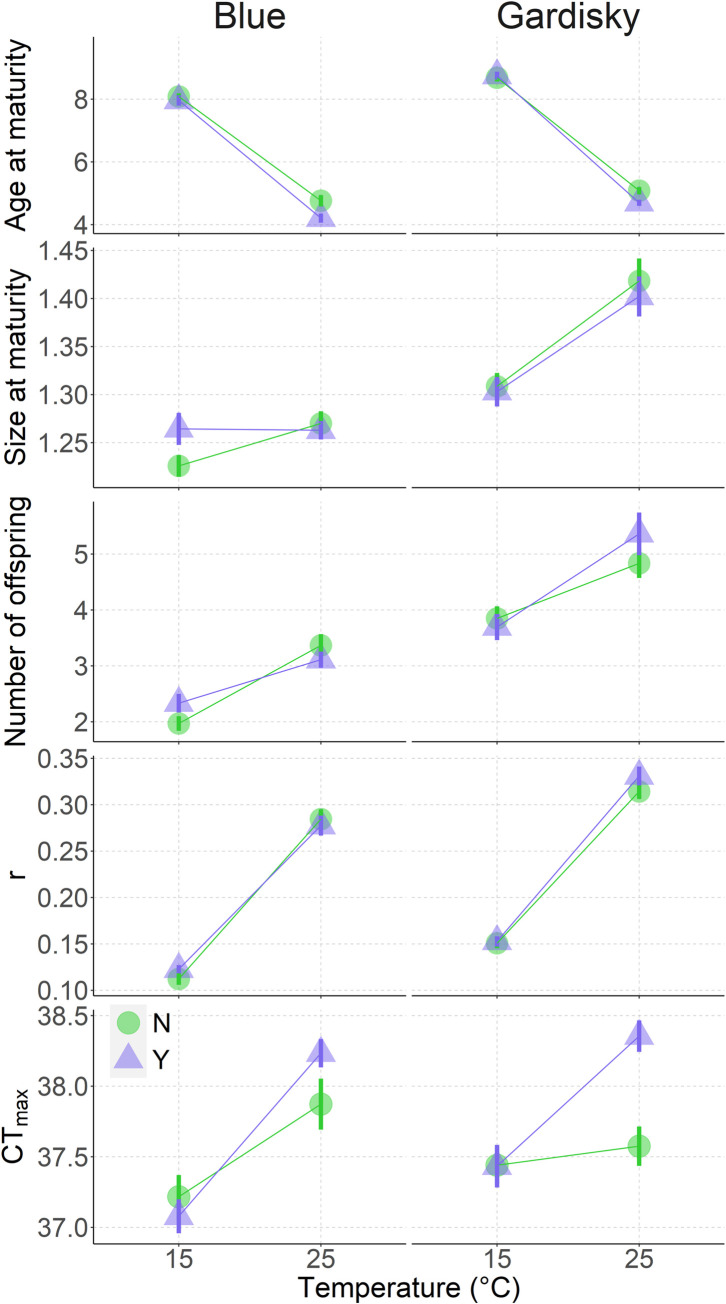
Predation cues influence critical maximum temperature of *Daphnia pulicaria*. Means ± 1 S.E.M. of age at maturity (days), size at maturity (mm), average number of offspring of the first three clutches, intrinsic growth rate (r), and critical maximum temperature (CTmax, °C) of Daphnia pulicaria collected at Blue and Gardisky Lakes during summer 2017 in response to temperature (15°C and 25°C, x-axis) and predation cues treatments. Circles show response without fish cues (N) and triangles show response to fish cues (Y).

Several traits in the two clones responded differently to temperature and fish cues (Table 1). Age at maturity was earlier for both clones in the presence of fish cues at high temperature (Temperature x Fish cues: p-value = 0.007, Table 1, Fig 1). The post-hoc test (least square means) showed that fish had an effect only on the Blue Lake clone reared at 25°C. At 25°C, Blue Lake replicates reared with fish cues matured 0.55 ± 0.16 days earlier compared to replicates without fish cues (Fig 1).

**Table 1:** Fixed-effect results of the general linear mixed-effect model analysis of the life-history traits of *Daphnia pulicaria*. Clonal lines were collected from Blue and Gardisky Lakes and reared under different temperature (15°C or 25°C) and fish cue treatments (presence or absence). A clone’s lake of origin was treated as a random effect.

Factor	Estimate	Std. Error	df	T	Pr(>|t|)
**Age at maturity**					
(Intercept)	2.123844	0.047833	1.234098	44.401	**0.006**
Temperature	-0.5418	0.024793	181.0195	-21.853	**<0.001**
Fish cues	-0.0059	0.024384	181.0032	-0.242	0.809
Temperature x Fish cues	-0.09471	0.034685	181.0142	-2.731	**0.007**
**Size at maturity**					
(Intercept)	0.23547	0.03907	1.07948	6.027	0.092
Temperature	0.05783	0.01244	179.007	4.65	**<0.001**
Fish cues	0.01247	0.01236	179.0011	1.009	0.314
Temperature x Fish cues	-0.02189	0.01749	179.005	-1.252	0.212
**Average number of offspring**					
(Intercept)	0.99243	0.25649	1.05045	3.869	0.152
Temperature	0.37875	0.06529	84.00142	5.801	**<0.001**
Fish cues	0.05975	0.06611	84.00343	0.904	0.369
Temperature x Fish cues	-0.04401	0.09203	84.00474	-0.478	0.634
**Intrinsic growth rate**					
(Intercept)	0.131451	0.019855	1.143028	6.62	0.075
Temperature	0.167526	0.008244	84.00395	20.32	**<0.001**
Fish cues	0.005761	0.008348	84.00951	0.69	0.492
Temperature x Fish cues	-0.00052	0.011621	84.01312	-0.045	0.964
**Critical maximum temperature (CT**_**max**_ **)**					
(Intercept)	3.619419	0.002787	12.85123	1298.746	**<0.001**
Temperature	0.010592	0.003818	89.30079	2.775	**0.007**
Fish cues	-0.00205	0.003708	89.00375	-0.552	0.582
Temperature x Fish cues	0.017227	0.005291	89.09375	3.256	**0.002**

Temperature affected size at maturity in both populations (Temperature: p < 0.001, Table 1) but fish cues had no main effect across clones (Fish: p = 0.314, Table 1, Fig 1). Post hoc tests revealed that the Blue Lake clone matured at 1.26 ± 0.006 mm regardless of temperature, while Gardisky matured at 15°C with 1.31 ± 0.01 mm compared to 1.41 ± 0.01 mm at 25°C.

For the Blue Lake clone, offspring number only varied with temperature; on average Blue had 1.1 ± 0.11 more offspring at 25°C compared to 15°C. In contrast, the effect of temperature on fecundity was greater for the Gardisky Lake clone when fish cues were present. Individuals had 1.67 ± 0.30 more offspring at 25°C than 15°C in the presence of fish cues, while without fish cues the difference between 25°C and 15°C response was 0.98 ± 0.23 offspring.

The intrinsic growth rate was significantly affected by the main effects of temperature but not predation (Temperature: p < 0.001, Table 1, Fig 1). At each temperature, the Gardisky Lake clone had higher intrinsic growth rates compared to the Blue Lake clone. For instance, the Gardisky clone had an *r* = 0.15 ± 0.001*day^-1^ at 15°C, while Blue had *r* = 0.11 ± 0.004*day^-1^. At 25°C, the intrinsic growth rate was 0.32 ± 0.006*day^-1^ for the Gardisky Lake clone, while the growth rate for the Blue Lake clone was 0.28 ± 0.007*day^-1^.

CT_max_ increased significantly when *Daphnia* were reared at 25°C compared to 15°C. CT_max_ was significantly affected by the interaction between temperature and fish cues (Temperature x Fish cues: p = 0.002, Table 1). Post hoc tests showed that at 15°C, fish cues had no effect on CT_max_. The thermal tolerance of the Gardisky clone increased by 0.8 ± 0.12°C in the presence of fish cues, while the Blue Lake clone increased by 0.3 ± 0.14°C at 25°C.

### Gene expression

**Fig 2:**
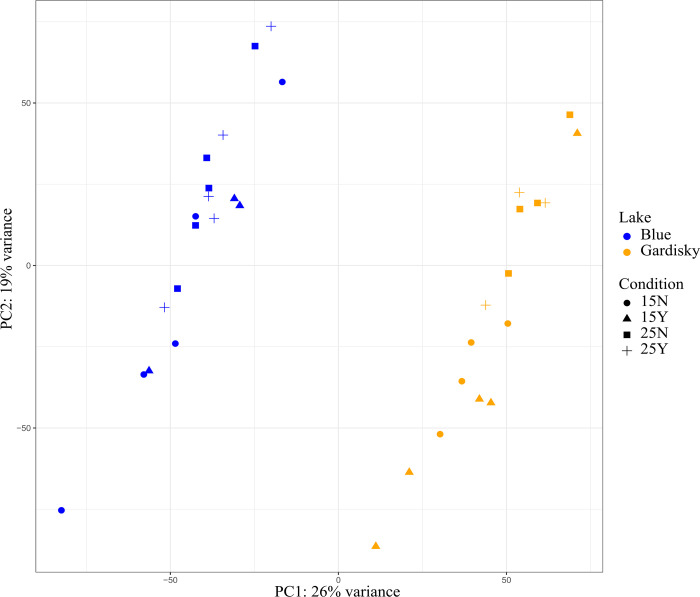
Genotype, not treatment group, is the dominant source of differential gene expression. Principal component analysis of gene expression patterns generated through DESeq2 prominently separates individuals by clone along the first principal component.

For the Blue Lake clone, sequencing produced on average 42,306,315 raw pair-ended reads for each sample. Across all Blue Lake samples, 79.4% of the cleaned reads (after removing adaptor sequences, low-quality and ambiguous sequences) were mapped to the D. pulicaria LK16 primary gene set. The Gardisky clone produced on average 44,025,859 raw pair-ended reads for each sample and 80.0% of the cleaned reads mapped to the D. pulicaria LK16 primary gene set. A breakdown of read mapping rate and estimated read set contamination by sample is visualized in S1 Fig. *De novo* assemblies of the Blue and Gardisky clones show sequence similarity to previously annotated *Daphnia* genomes (S2 Fig) and high levels of completeness consistent with previously annotated arthropod genomes (S3 Fig). A PCA plot generated using DESeq2-corrected read counts (Fig 2) shows clear divergence in the gene expression between clones under all growth conditions. A PERMANOVA test of gene expression data shows that gene expression was affected by both clone (Pseudo F = 9.6582, R^2^ = 0.22284, p = 0.001) and temperature treatments (Pseudo F = 4.367, R^2^ = 0.10078, p = 0.001), but was not significantly different between predation treatments (Pseudo F = 0.6706, R^2^ = 0.01547, p = 0.746).

**Fig 3:**
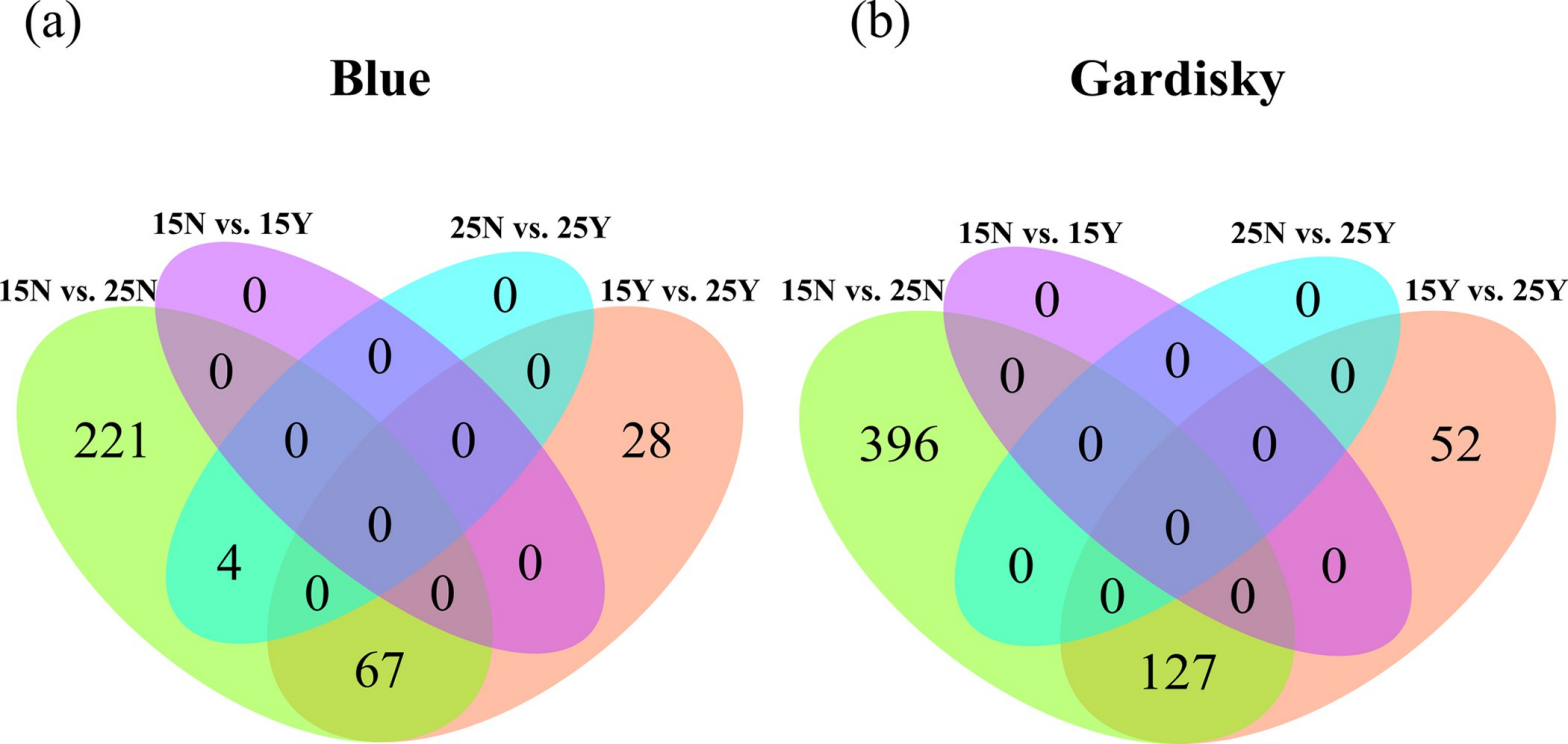
Many genes are differentially expressed in response to temperature changes, while kairomones have minimal observable effect at the level of transcript expression. Venn diagram of differentially expressed genes for *Daphnia pulicaria* clones **(a)** Blue and **(b)** Gardisky in response to temperature and predation cues. Cyan and purple sections illustrate differential expression from the introduction of predator cues (Y vs. N for the presence and absence of fish cues), while the green and orange sections illustrate differential expression due to temperature change. Treatment abbreviations: 15Y, 15°C with kairomones; 15N, 15°C without kairomones; 25Y, 25°C with kairomones; 25N, 25°C without kairomones.

Temperature treatments (25N vs. 15N and 25Y vs. 15Y) produced the greatest number of differentially expressed genes (DEGs) for both the Blue and Gardisky clones (Fig 3). A total of 320 genes were differentially expressed between one-factor comparisons for the Blue Lake clone: 95 due to temperature differences with predation cues, 292 due to temperature differences without predation cues, and 4 due to fish cues at 25°C (Fig 3A). For the Gardisky Lake clone, we identified 575 DEGs. In total, 396 DEGs were specific to 25N vs. 15N and 52 DEGs were specific to 25Y vs. 15Y. These two treatment comparisons shared 127 genes (Fig 3B). Predator cues alone had no measurable effect on gene expression when comparing between Gardisky clones grown under the same temperature. Tables listing all differentially expressed genes are presented in S1A and S1B Table.

Comparisons of significant GO terms for differentially expressed genes were difficult to interpret due to the limited specificity of the functional information they provide. Genes related to proteolysis, chitin turnover, and vision appeared to be upregulated at higher water temperatures, but no significantly over- or under-represented GO terms were detected in response to kairomones. GO terms for phototransduction and visual perception were enriched in all temperature comparisons, while protease and peptidase activity were enriched in every comparison but 25Y vs. 15Y in the Blue clone. Meanwhile, terms associated with chitin binding were enriched in every comparison but 25Y vs. 15Y in the Gardisky clone. Complete lists of all enriched GO terms are given as S2A–S2D Table.

### Gene orthology

**Fig 4:**
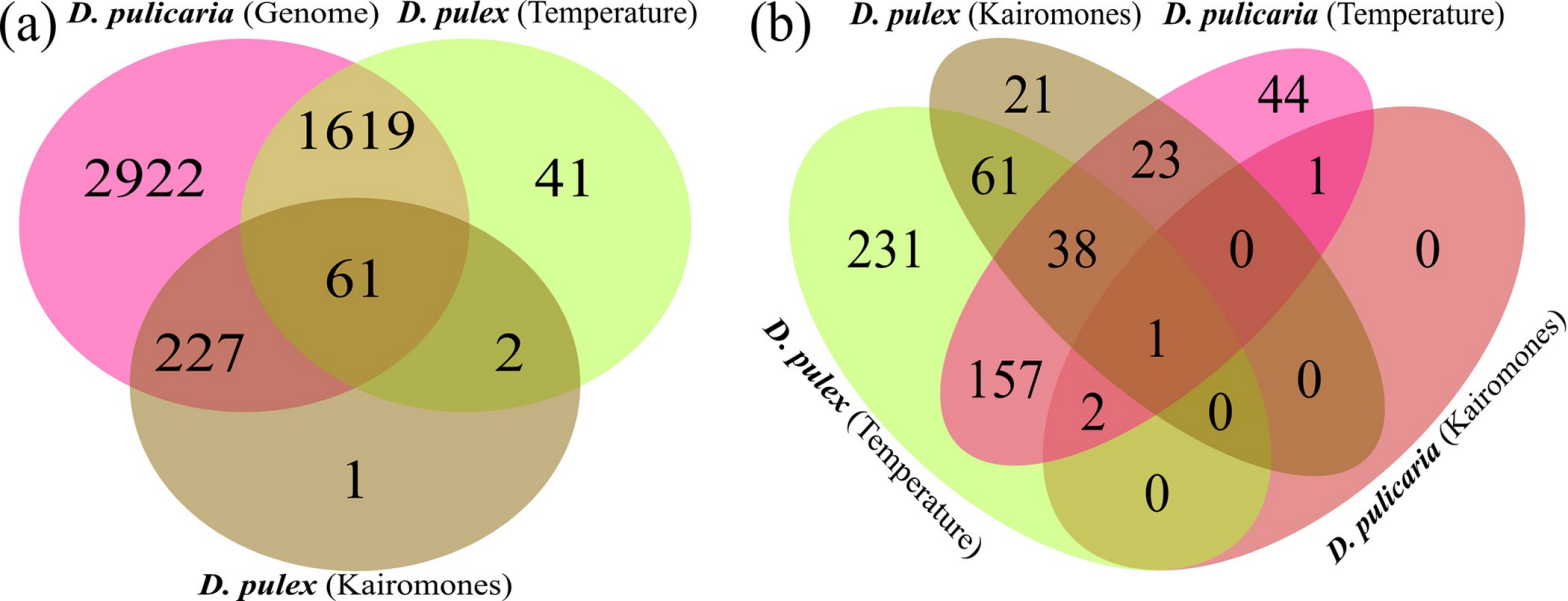
Gene orthology analysis reveals similarities between stress-responsive genes in *D*. *pulicaria* and *D*. *pulex*. Venn diagrams of orthologous gene clusters between *D*. *pulex* DEGs from the *Daphnia* Stressor Database and **(a)** the *D*. *pulicaria* genome or **(b)** DEGs from *Daphnia pulicaria*. Numbers indicate protein families with at least two members, excluding "singletons" that have no orthologous or paralogous matches. Gene identifiers for orthologous clusters and singleton genes are available in **(a)** S1 File and **(b)** S2 File.

From the Daphnia Stressor Database, we retrieved 386 genes that responded to kairomones in *D*. *pulex* across 7 studies, and 2660 genes that responded to temperature changes in *D*. *pulex* across 4 studies. 315 of these previously documented kairomone-responsive genes (81.6%) have orthologs in the *D*. *pulicaria* LK16 genome, while 1796 of the temperature-responsive genes (67.5%) are orthologous to genes in *D*. *pulicaria* (Fig 4A). 35% of the temperature responsive *D*. *pulicaria* DEGs in this study are orthologous to previously reported temperature-responsive genes from *D*. *pulex*, but only one of the four genes in the Blue clone that responded to kairomones was orthologous to a previously described kairomone-sensitive gene (Fig 4B). On the other hand, we observed that 18% of described kairomone-responsive genes in *D*. *pulex* have orthologs that respond to temperature in *D*. *pulicaria*, consistent with earlier phenotypic observations (Fig 1) showing an interactive relationship between these variables. Lists of gene identifiers for orthologous clusters and singleton genes are available in S1 and S2 Files.

## Discussion

*Daphnia* clonal lines that were stressed by temperature and/or predator cues exhibited similar phenotypic responses associated with distinctly different patterns of gene regulation. Temperature was the most important factor driving changes in gene expression, as most of the DEGs in both clones responded to this variable, versus only a handful to kairomone cues. Yet fish cues magnified the effect of 10°C of warming on age of maturity and increased the critical maximum temperature at which *D*. *pulicaria* lose motor control when individuals were reared at high temperatures, but not at their optimum temperature. Genetic pathways that underlie the plastic response to predators and temperature may be subject to different regulatory pressures, but phenotypic responses suggest that exposure to predator cues may increase thermal tolerance and affect life history response to warming.

### Phenotypic responses

Both clones matured at a younger age and produced more offspring when reared in high temperature, thus having a higher intrinsic growth rate compared to lower temperature conditions (Fig 1). Indeed, high temperature increases metabolic rate, often leading to a larger reproductive investment and faster development [59, 60]. In addition, we found an interactive effect of temperature and predation on age at maturity and critical maximum temperature.

Our finding that individuals reared with predator cues had higher thermal tolerance and matured younger at 25°C than those reared without predator cues agree with other studies that also found synergistic effects between these two stressors in *Daphnia* and other aquatic organisms [61–66]. In *D*. *pulex*, after seven generations, Tseng and O’Connor observed increased thermal plasticity in individuals reared in higher temperatures only when they were also reared with predators [61]. Zhang et al. tested resurrected *D*. *magna* population from time periods without fish and from a high-density fish period [62]. They found that *Daphnia* that coexisted with fish exhibited earlier maturation in high temperatures compared to *Daphnia* from the pre-fish period. Earlier maturation under predation risk is common in size-dependent predation of *Daphnia* where predators, such as fish, prefer larger prey items, inducing a smaller size at maturity and earlier age of first reproduction [60]. Our results agree with these studies showing that fish cues magnify the life history response to temperature and show that exposure to fish predation can increase the tolerance of stressfully high temperatures.

### Genetic responses to temperature

We observed upregulation of serine peptidases in response to high temperature treatment for both clones. These enzymes are the most important digestive proteases in *D*. *magna* [67]. They are involved in proteolysis in the digestive system, the process by which peptide bonds in proteins are broken generating free amino acids. This upregulation may indicate a necessity to accommodate higher feeding rates caused by increased metabolism in high temperatures [59, 60]. Moreover, the *D*. *pulex* genome contains many peptidase genes, which might indicate adaptation to high variation in food availability in aquatic environment [68].

Genes related to visual perception were also upregulated in response to high temperature treatment in both clones. Vision has previously been associated with vertical migration, because visible light is a proxy for both *Daphnia’s* visibility to predators and the amount of incoming photodamage due to UV radiation at near-surface depths [69]. Vertical migration can serve as either a predator avoidance mechanism induced directly from kairomones or a diel behavior triggered by temperature change [69]. Higher temperatures can serve as a proxy for shallower depths, and therefore more incoming radiation. This may explain why our dataset shows enrichment of phototransduction genes as a response to temperature while previous studies have observed phototransduction gene enrichment in response to kairomones [28].

### The missing kairomone response: Behavioral changes and proximate cues

In total, we only detected 4 DEGs across two *Daphnia pulicaria* clones in response to kairomones from *Oncorhynchus mykiss*. Such a low number of DEGs related to kairomone exposure is unexpected, but not unprecedented in previous *Daphnia* studies. One study on *Daphnia galeata* M6 by Tams et al. also found 4 differentially expressed transcripts in response to kairomones from the fish *Leuciscus idus* using a two-factor analysis [70], and another study found no DEGs in *Daphnia magna* Iinb1 when exposed to kairomones from stickleback fish [71]. Tams et al. hypothesize that life history changes might be associated with only a few genes compared to morphological defenses, or that regulation could be post-translational and therefore would not appear in an RNA sequencing study [70]. Defense strategies can also include behavioral changes such as vertical migration [72, 73] and the transition into a state of alertness [74], which RNA expression data may not fully capture. In contrast, morphological changes such as neckteeth formation have been associated with far more genes, such as 230 DEGs found in *D*. *pulex* in response to *Chaoborus* kairomones [75].

Miehls et al. find that the spiny water flea *Bythotrephes longimanus* displays no changes in life history or morphology when exposed to kairomones from the predator *Perca flavescens*, but increased temperature does induce morphologic defense against predation [76]. The authors hypothesize that temperature is a stronger predictor of predation risk than kairomones. This is because adult *P*. *flavescens* are present year-round but only juveniles are gape-limited in their consumption of *B*. *longimanus*, such that the morphologic response of increased tail length is only effective in discouraging predation from juveniles and not adult fish. If there is no difference between the kairomones released from juvenile and adult *P*. *flavescens*, then temperature could serve as a proximate cue for predation risk that could be damped through some phenotypic response.

The interactions between *B*. *longimanus* and *P*. *flavescens* closely mirror predation of *D*. *pulicaria* by *O*. *mykiss*, as *O*. *mykiss* are among the most abundant stocked fished in the Sierra Nevada lakes [77] and thus *D*. *pulicaria* can be exposed to their kairomones year-round. Juvenile *O*. *mykiss* prey on *Daphnia* as a large percentage of their diet, while trout larger than 350 mm are primarily piscivorous [25]. A morphological defense response targeted solely at juvenile trout might require either a juvenile-specific odor, a dynamic response based on cue concentration, or some additional proxy for *O*. *mykiss* life stage that would suggest a high concentration of juveniles. Riessen and Gilbert propose that *D*. *pulex* may use water temperature to serve as a proximate cue for predator densities [78], so water temperature as a proxy for predator life stage is not implausible. Our dataset found some overlap between kairomone-responsive genes in *D*. *pulex* and temperature-responsive genes in *D*. *pulicaria*, so an intermediate defensive response may be inducible through heat alone as found by Suppa et al. [79].

However, behavior-based predator avoidance strategies may not require such specificity. *O*. *mykiss* can produce the kairomone 5α-cyprinol sulfate in their bile without consuming *Daphnia*, and this compound induces diel vertical migration in *D*. *pulex* [72]. Vertical migration still has an associated tradeoff in fitness [80], but this defense might be preferred when food sources are abundant and temperature gradients are tolerable [81]. Another study found that *D*. *pulicaria* lineages that coexist with *O*. *mykiss* vertically migrate without kairomone exposure, while *D*. *pulicaria* lineages without prior contact with *O*. *mykiss* require kairomone exposure to induce diel vertical migration [82]. Thus, some environments with high predation might select for *Daphnia* genotypes that respond to predation primarily through behavioral responses, such as vertical migration.

### The missing kairomone response: Predator- and diet-specific cues

An alternative explanation for the lack of DEGs may lie in the subtle differences between kairomone sources. Some kairomones are innately released by the predator (predator odors), from damaged prey (alarm cues), and from digested prey excreted by the predator (dietary cues). This study and Tams et al. explicitly avoided the addition of conspecific alarm cues in our kairomone mixture as to measure only the effect of predator odors [70]. Other studies, such as [27, 28], tested responses to a mixture of predator-specific odors and alarm cues released by consumed conspecifics. Mitchell et al. call for the separation of nomenclature between cues released from the predator regardless of diet and diet-specific cues [29], so that researchers can more accurately discern the independent effects of these different kairomone compounds.

Previous research on the interaction between predator odors and alarm cues is mixed. Some studies propose that defense systems rely on both types of compounds to induce measurable defense responses [83, 84], while others observe that alarm cues by themselves can induce separate responses than predator odors alone [85, 86]. Alarm cues are thought to be nonspecific, as *Daphnia* respond differently to different predators [87] and alarm cues alone might be unable to induce defense responses effective against a specific predator [88]. The most effective defense responses may therefore require predator odors to identify the type of predator combined with alarm cues to induce an immediate response. A required mixture of predator odors and alarm cues for maximum observed response in *D*. *pulicaria* would succinctly explain why we observed such a minor response in our experiment. Results from this study support the conclusion that the detection of predator odors alone may be insufficient to induce defense responses at the level of gene expression, at least in the context of *D*. *pulicaria–O*. *mykiss* interactions.

## Conclusion

Our study highlights that expression patterns of genes differed between *Daphnia pulicaria* clones while phenotypic responses and interactions were qualitatively similar, suggesting that different transcriptomic responses can result in similar phenotypes. Other studies have found distinct gene expression patterns among individuals from different zooplankton species [89, 90]. These results suggest that diverse genetic pathways can give rise to similar phenotypic plastic responses to environmental stress. We also showed synergistic interactions between temperature and predation for some traits. Our results show that *Daphnia* can have similar phenotypes through distinct molecular mechanisms and the synergistic effects of temperature and predation may represent an important mechanism for organisms to adapt to a rapidly changing environment. As one of the first studies incorporating the *Daphnia pulicaria* genome, we hope to lay the groundwork for future differential expression studies through incorporation into knowledge bases such as the *Daphnia* Stressor Database. Overall, we find that the interactions between stressors are heavily genotype dependent, and echo the sentiment that novel research strategies are necessary [29] to further investigate the chemical signals of predation in *Daphnia*.

## Supporting information

S1 Table Data associated with Fig 3. Differentially expressed genes in the **(a)** Blue Lake clone and **(b)** the Gardisky Lake clone. The comparison column denotes if genes were responsive to temperature and/or kairomone treatments.
(XLSX)

S2 Table Gene Ontology of differentially expressed genes. Upregulated Gene Ontology terms for comparisons between: **(a)** Blue Lake clone, 15Y and 25Y treatments, **(b)** Blue Lake clone, 15N and 25N treatments, **(c)** Gardisky Lake clone, 15Y and 25Y treatments, and **(d)** Gardisky Lake clone, 15N and 25N treatments.
(XLSX)

S1 Fig Read-based taxonomy assignment consistent with mapping rates to the *Daphnia pulicaria* genome. Panel **(a)** shows Kraken2 read taxonomy on a random subset of one million cleaned reads from each sample. Panel **(b)** plots the alignment rate of the cleaned reads from each sample to the chromosomes of *Daphnia pulicaria* LK16 using the STAR aligner.
(TIF)

S2 Fig Blue and Gardisky transcripts are highly similar to *Daphnia pulicaria* LK16. Results of a two-way average nucleotide identity (ANI) matrix and associated dendrogram for *the de novo* assembled transcriptomes from Blue and Gardisky Lakes, predicted genes from *Daphnia* species with annotated genomes, and the annotated gene set of the related arthropod *Eulimnadia texana*. Our transcriptome sets are closest to those from *D*. *pulicaria*, within the well-described *Daphnia pulex-pulicaria* species complex.
(TIF)

S3 Fig Blue and Gardisky transcriptomes are highly complete and highly duplicated. Bar graph of gene set completeness as determined by BUSCO using the arthropod marker gene set. Our *de novo* assembled transcriptomes have completeness scores comparable to or better than genome-derived transcript sets from other *Daphnia* studies. However, Blue and Gardisky transcriptome sets have additional gene duplication in our transcriptome due to the inclusion of gene isoforms from *de novo* assembly.
(TIF)

S1 File Orthologous clusters associated with Fig 4A. Orthologous clusters and singletons for the gene orthology analysis between the *D*. *pulicaria* genome, temperature-responsive genes in *D*. *pulex*, and kairomone-responsive genes in *D*. *pulex*. *D*. *pulex* genes were collected from the *Daphnia* Stressor Database. Data is in Excel format.
(XLSX)

S2 File Orthologous clusters associated with Fig 4B. Orthologous clusters and singletons for the gene orthology analysis between temperature-responsive *D*. *pulicaria* genes, kairomone-responsive *D*. *pulicaria* genes, temperature-responsive genes in *D*. *pulex*, and kairomone-responsive genes in *D*. *pulex*. *D*. *pulex* genes were collected from the *Daphnia* Stressor Database. Data is in Excel format.
(XLSX)
