# Supplementary figures and images for: Phenotypic and transcriptional response of Daphnia pulicaria to the combined effects of temperature and predation
Source: PLoS One. 2022 Jul 14;17(7):e0265103. doi: 10.1371/journal.pone.0265103 (PMC9282536; doi:10.1371/journal.pone.0265103)

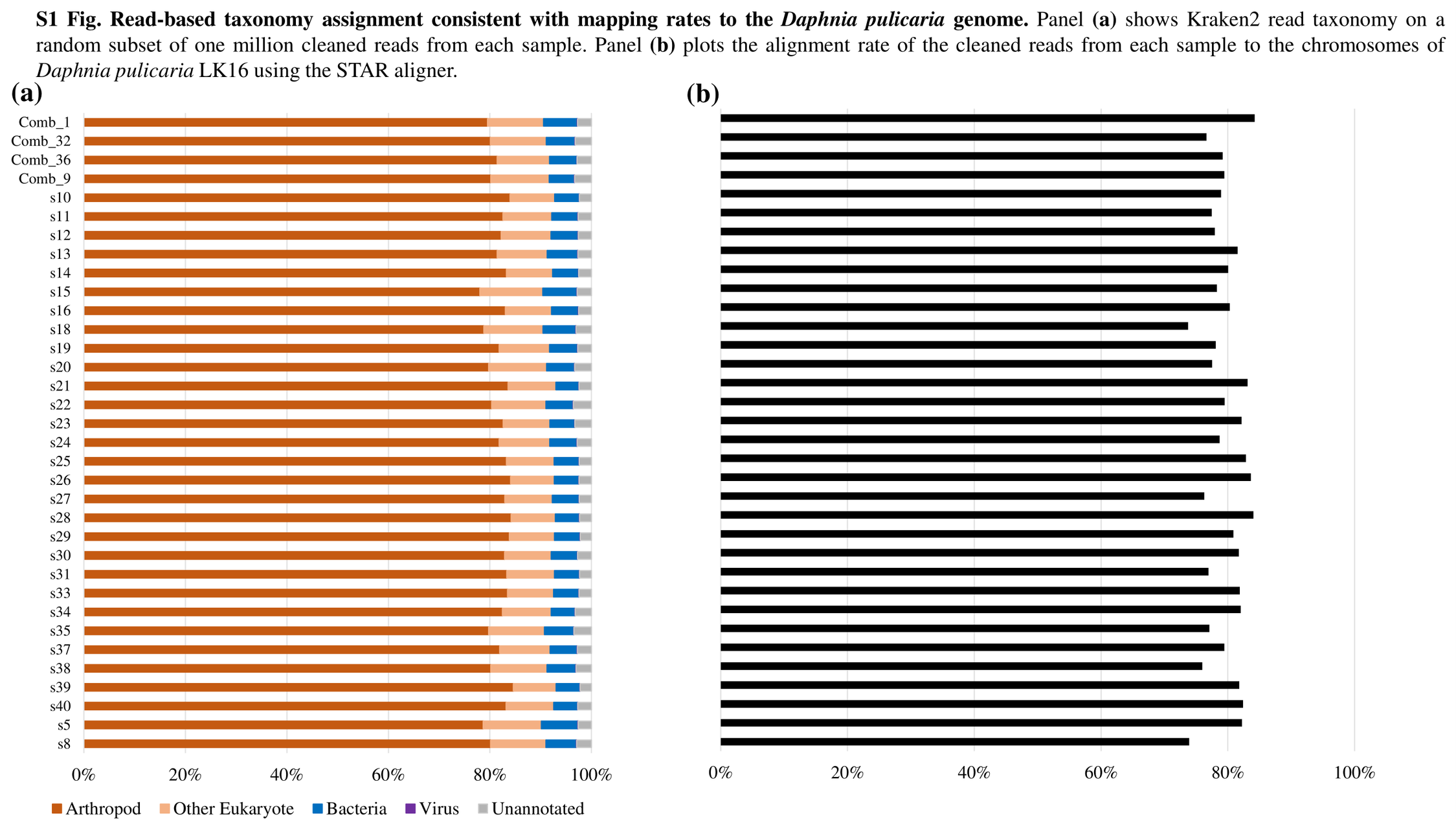

Supplement: S1 Fig — Panel (a) shows Kraken2 read taxonomy on a random subset of one million cleaned reads from each sample. Panel (b) plots the alignment rate of the cleaned reads from each sample to the chromosomes of Daphnia pulicaria LK16 using the STAR aligner. (TIF) [file pone.0265103.s003.tif]

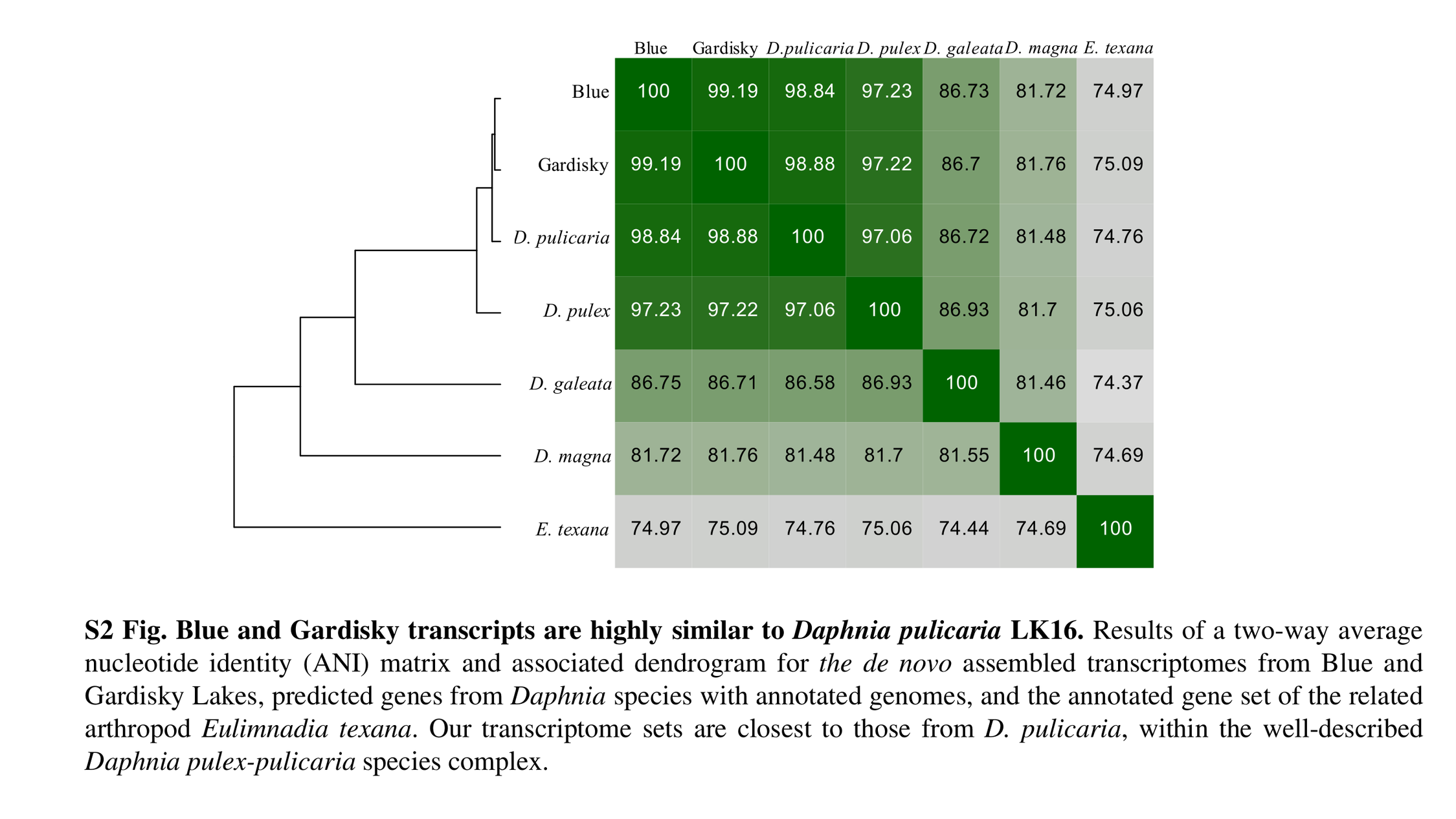

Supplement: S2 Fig — Results of a two-way average nucleotide identity (ANI) matrix and associated dendrogram for the de novo assembled transcriptomes from Blue and Gardisky Lakes, predicted genes from Daphnia species with annotated genomes, and the annotated gene set of the related arthropod Eulimnadia texana. Our transcriptome sets are closest to those from D. pulicaria, within the well-described Daphnia pulex-pulicaria species complex. (TIF) [file pone.0265103.s004.tif]

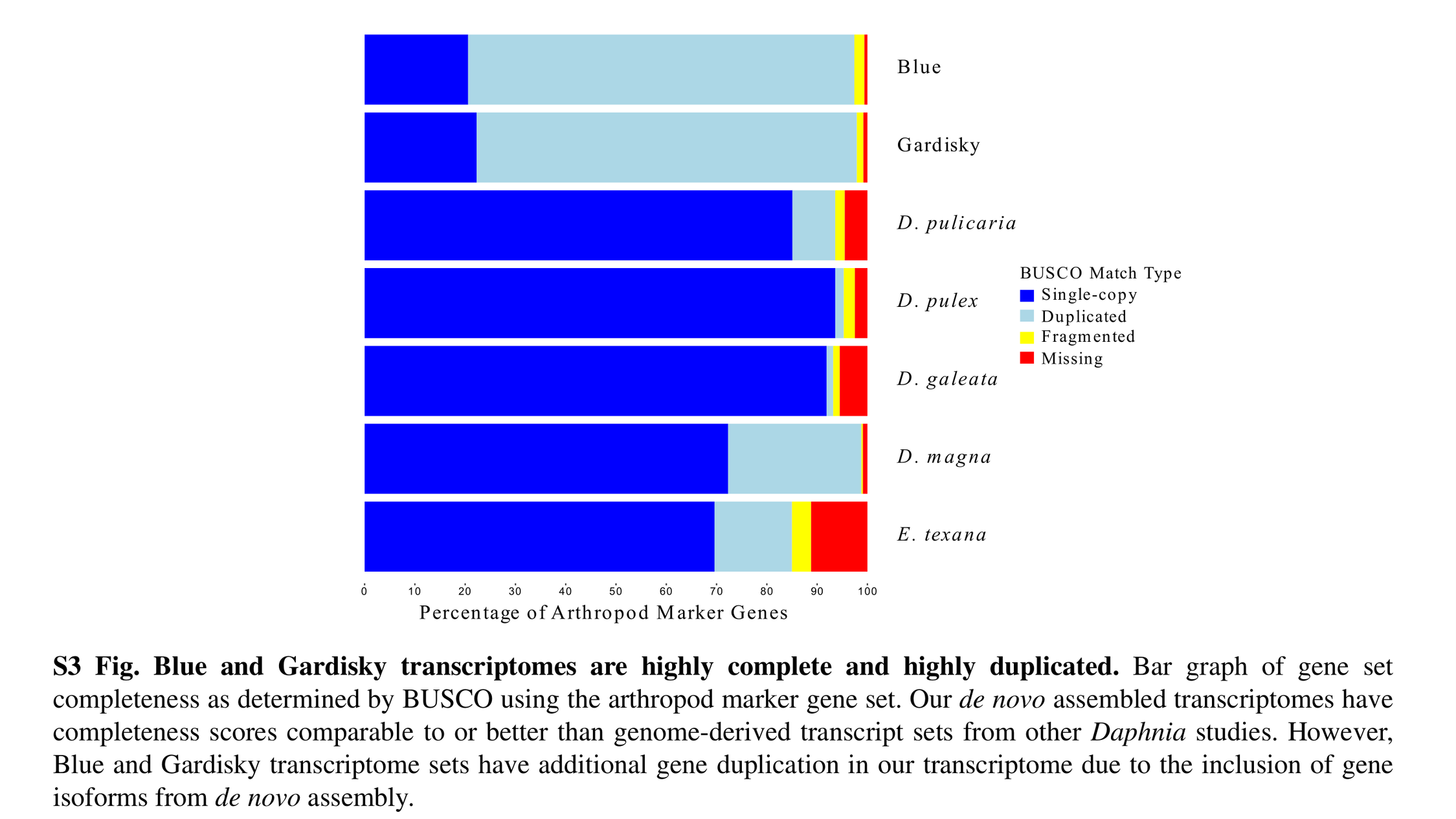

Supplement: S3 Fig — Bar graph of gene set completeness as determined by BUSCO using the arthropod marker gene set. Our de novo assembled transcriptomes have completeness scores comparable to or better than genome-derived transcript sets from other Daphnia studies. However, Blue and Gardisky transcriptome sets have additional gene duplication in our transcriptome due to the inclusion of gene isoforms from de novo assembly. (TIF) [file pone.0265103.s005.tif]
